# Logistics-Mediated Artificial Sympatry and Its Implications for Molecular Detection of *Hylurgus ligniperda*

**DOI:** 10.3390/insects17040408

**Published:** 2026-04-09

**Authors:** Jijing Han, Jiaying Wang, Junxia Cui, Li Liu, Xianfeng Chen, Yuhao Cao, Jiaojiao Chen, Xuemei Song

**Affiliations:** 1 State Key Laboratory for Quality and Safety of Agro-Products, Key Laboratory of Biotechnology in Plant Protection of MARA, Key Laboratory of Green Plant Protection of Zhejiang Province, Institute of Plant Virology, Ningbo University, Ningbo 315211, China; hanjijing2023@163.com (J.H.); caoyuhao@nbu.edu.cn (Y.C.); chenjiaojiao@nbu.edu.cn (J.C.); 2 Technical Center, Ningbo Customs, Ningbo 315100, China; wangjy877@163.com (J.W.); flyingcjx@163.com (J.C.); 3 Ningbo Zhongsheng Product Testing Co., Ltd., Ningbo 315048, China; liuli545@126.com; 4 Ningbo Customs, Ningbo 315012, China; cxfxhy17@163.com

**Keywords:** *Hylurgus ligniperda*, cross-reactivity, invasive forest pests, molecular diagnostics, biosecurity

## Abstract

International timber trade has accelerated the global spread of the invasive red-haired pine bark beetle *H. ligniperda*, posing persistent challenges to phytosanitary inspection and border biosecurity. Rapid isothermal amplification assays are increasingly deployed in frontline quarantine settings to support timely regulatory decisions. However, their performance under the heterogeneous biological backgrounds typical of traded timber remains insufficiently evaluated, particularly with respect to the practical implications of low-level false-positive signals. We re-evaluated a previously reported isothermal assay for *H. ligniperda* using conditions that simulate timber transport and routine customs workflows. Fifty non-target arthropod species (predominantly insects), selected from quarantine interception records, were included to represent taxa likely to co-occur in operational contexts. Material from *Lema decempunctata* consistently generated weak but reproducible amplification signals across replicates. Sanger sequencing excluded contamination, confirming low-level non-target amplification in complex biological matrices. Although the signals were faint, ambiguous results in quarantine settings may trigger shipment detention, confirmatory laboratory testing, or temporary trade restrictions, thereby increasing inspection workload, delaying clearance, and generating avoidable compliance costs. These findings indicate that trade-mediated species assemblages can compromise assay performance beyond expectations derived from conventional taxonomy-based specificity testing. To reduce interpretive uncertainty and associated regulatory burden, we propose a tiered diagnostic workflow combining rapid on-site isothermal screening with specificity-oriented SYBR Green qPCR confirmation. This strategy enhances diagnostic reliability while preserving operational efficiency in applied biosecurity surveillance.

## Simple Summary

This study reveals the limitations of relying solely on taxonomy-based specificity testing for molecular assays in complex quarantine and trade scenarios. Isothermal amplification assays can produce low-intensity, non-target signals due to probabilistic primer–template interactions and the mixing of biological materials during logistics. Such faint false positives, though infrequent, may trigger unnecessary regulatory actions, including shipment detention, confirmatory testing, and trade delays, thereby increasing operational burdens and costs. To mitigate interpretive uncertainty and enhance reliability, we recommend a tiered diagnostic approach: rapid on-site isothermal screening followed by specificity-focused SYBR Green qPCR confirmation. These findings underscore the importance of developing risk-oriented validation frameworks that better reflect real-world trade conditions to support efficient and robust pest surveillance in applied biosecurity settings.

## 1. Introduction

*Hylurgus ligniperda* (Fabricius) (Coleoptera: Curculionidae: Scolytinae), commonly known as the red-haired pine bark beetle, is a quarantine-regulated invasive forest pest affecting pine-producing regions worldwide [1,2,3,4]. Its spread has been closely associated with the international trade of raw timber and solid wood packaging materials. Continued expansion of global wood commerce has increased opportunities for transboundary introduction and establishment [5,6,7,8,9,10]. In this setting, reliable molecular diagnostic tools are essential for early detection and quarantine decision-making [11,12,13,14].

Isothermal amplification technologies, including multiple enzyme isothermal rapid amplification (MIRA), recombinase-aided amplification (RAA), and recombinase polymerase amplification (RPA), are increasingly applied in field-based pest diagnostics due to their rapid amplification kinetics and limited equipment requirements [15,16,17,18,19,20,21,22,23,24]. Previous studies have reported high analytical sensitivity and operational feasibility for target identification [25,26,27,28]. Specificity testing has generally focused on phylogenetically related taxa, based on the assumption that non-target amplification is most likely among closely related species [29].

Inspection samples associated with contemporary trade may contain mixed biological materials that do not reflect natural ecological assemblages. Commodities from multiple production regions are commonly aggregated at ports and logistics hubs [10,30]. Under these circumstances, organisms with distinct ecological niches may co-occur during transport and handling. Such situations can introduce additional biological background into diagnostic procedures.

**Figure 1:**
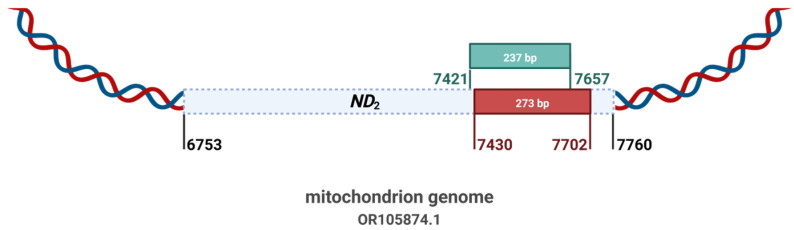
Target region comparison and primer design for qPCR confirmation of *H. ligniperda*.

To examine assay performance under these conditions, we conducted an expanded exclusivity assessment of a previously reported isothermal amplification assay for *H. ligniperda* [15] (Figure 1). Fifty non-target species were included, encompassing phylogenetic relatives, wood-associated taxa, and species likely to occur within logistical supply chains. Among these, only the phylogenetically distant leaf beetle *Lema decempunctata* (Gebler) produced reproducible low-intensity amplification signals. The two species differ substantially in ecology and host association: *L. decempunctata* primarily exploits Solanaceae hosts, whereas *H. ligniperda* colonizes pine timber [31,32,33,34,35,36,37] (Figure S1). Their potential co-occurrence in trade samples is therefore more plausibly attributable to commodity aggregation than to direct ecological interaction [38,39].

These observations suggest that assay validation under trade-associated conditions may provide additional information beyond conventional phylogeny-based specificity testing. Incorporating expanded exclusivity panels and clearly defined interpretation criteria may improve diagnostic robustness in applied quarantine and regulatory contexts [40].

## 2. Materials and Methods

### 2.1. Specimen Source and Nucleic Acid Preparation

Arthropod specimens, including *H. ligniperda*, were obtained from retained samples collected during routine border quarantine inspections at Beilun, Meishan, and Daxie ports by Ningbo Customs. *H. ligniperda* was treated as the target species. Fifty non-target arthropod species were included for exclusivity testing (Supplementary Table S1). The panel comprised Scolytinae species, additional Coleoptera representing different families, several species from other insect orders, and one arachnid. The selection aimed to cover both phylogenetically related taxa and species that may be encountered in trade-associated inspection samples.

Specimens were identified morphologically and, where necessary, confirmed by mitochondrial COI sequencing. Genomic DNA (gDNA) was extracted individually using a commercial animal tissue kit (Simgen, 3101050, Hangzhou, China) following the manufacturer’s protocol. DNA concentration and purity were checked by spectrophotometry and agarose gel electrophoresis. DNA extractions with A260/280 ratios between 1.8 and 2.0 were retained. For comparability across taxa, DNA concentrations were adjusted to 40–50 ng·μL^−1^ and stored at −20 °C until use.

In addition to purified gDNA, crude lysates were prepared for each specimen. Tissue was mechanically homogenized, followed by addition of 50 μL nucleic acid release reagent (Bigfish Taq, FT1011, Hangzhou, China) and repeated pipetting. Purified DNA and crude lysates were tested in parallel under the same amplification conditions. Two independently collected batches of *L. decempunctata* were included.

### 2.2. Isothermal Amplification and Signal Quantification

The assay examined here was originally reported as recombinase polymerase amplification (RPA). Based on the kit configuration used in the published study, the reaction chemistry corresponds to a multiple enzyme isothermal amplification (MIRA) format. Experiments were carried out under the published reaction conditions [15].

For comparison, the same primer and probe sequences were also evaluated using a commercial RPA kit. Primer and probe sequences were not modified. MIRA reactions were performed with the AMP-Future kit (WLN8210KIT, Weifang, China) following the published protocol. RPA reactions used the Bigfish Taq kit (IAK0202, Hangzhou, China). Amplification products were visualized with lateral flow strips (Tiosbio, JY0201, Beijing, China). Test-line intensity was quantified by quantum dot (QD) fluorescence measurement using the F11 handheld fluorescence immunochromatographic analyzer (Kreader, Beijing, China) [41].

Fluorescence intensity (*y*) was related to log_10_-transformed target-equivalent copy numbers per reaction (*x*) using exponential regression derived from plasmid dilution series (2.25 × 10^0^–10^6^ copies·reaction^−1^).

For MIRA: *y* = 1.6696e ^(0.9346*x*)^, *R*^2^ = 0.9678.

For RPA: *y* = 5.6325e ^(0.787*x*)^, *R*^2^ = 0.9856.

Regression models were based on three independent dilution experiments, each run in technical triplicate. Fluorescence values from test samples were converted using the respective regression equation.

Amplification observed in at least two of three technical replicates from non-target templates, with no signal in no-template controls, was recorded as false-positive. Selected products were purified and subjected to Sanger sequencing to verify amplicon identity; the sequencing results, including alignment with reference sequences, are provided in Supplementary Figure S1.

### 2.3. SYBR Green qPCR Confirmation

A SYBR Green-based qPCR assay targeting a region overlapping the isothermal detection locus was used as an independent confirmation. Primers were designed for high specificity and empirically optimized to reduce non-specific amplification. Melting curve analysis was performed in all runs. Primer sequences are detailed in Table 1. The genomic localization and sequencing validation of amplicons generated by the respective primers are depicted in Figure 1 and Figure S2.

Comparison of the published MIRA target region (red, 7430–7702 bp) and the SYBR Green qPCR region (green, 7421–7657 bp) within the *H. ligniperda* genome (GenBank OR105874.1). The overlapping regions ensure continuity and enable direct comparability between methods. Primer sequences are provided in Table 1.

Each 20 μL reaction contained 10 μL 2× qPCR Master Mix (Toroivd, QST-100P, Shanghai, China), 0.05 μM of each primer, 1 μL template (plasmid or purified genomic DNA), 1 μL amplification enhancer (Bigfish Taq, BFT2001, Hangzhou, China), and nuclease-free water. Thermal cycling consisted of 94 °C for 3 min; 45 cycles of 94 °C for 30 s, 51 °C for 30 s, and 72 °C for 30 s; followed by melting curve analysis (60–90 °C). Reactions were performed in triplicate.

### 2.4. Calibration, Sensitivity, and Exclusivity Assessment

Standard curves were generated using serial dilutions of plasmid DNA and adult *H. ligniperda* genomic DNA. Amplification efficiency (E) was calculated from the slope of the standard curve:

E = (10^(−1/slope)^ − 1) × 100%.

The analytical limit of detection (LOD) was defined as the lowest concentration yielding positive amplification in at least 95% of replicates across three independent experiments [42].

The same 50-species non-target panel was tested by qPCR for direct comparison with isothermal results.

Isothermal amplification served as the primary screening method under both purified DNA and crude lysate conditions. Samples producing amplification signals were further examined by qPCR. All assays were conducted in technical triplicate with appropriate controls included in each run.

## 3. Results

### 3.1. Cross-Platform Evaluation of Non-Target Amplification

**Figure 2:**
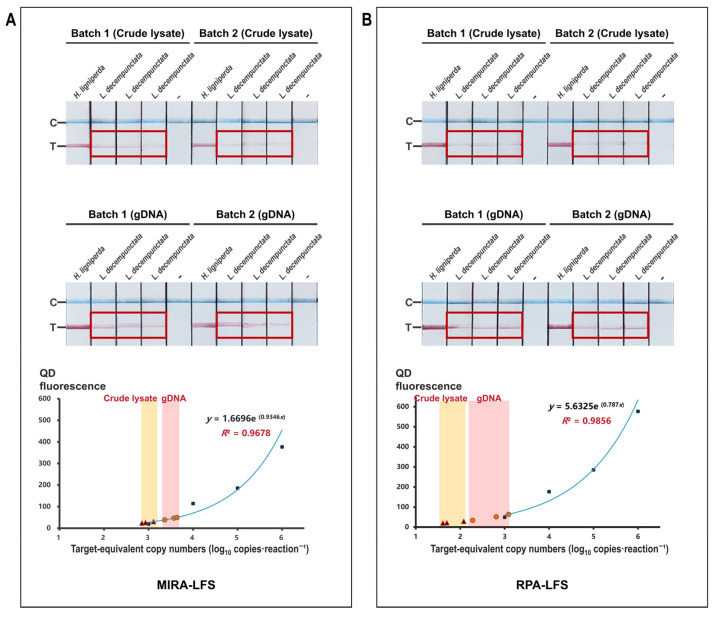
Cross-platform evaluation of T-line signals for *L. decempunctata.* (**A**) MIRA-LFS results showing reproducible signals in both crude lysates and purified genomic DNA across two independent biological batches. (**B**) RPA-LFS results showing comparable low-intensity signals. Three independent biological replicates were conducted for each platform; negative controls consisted of buffer or ultrapure water. Red boxes highlight consistent T-line signals across independent biological batches of *L. decempunctata*. In the fitted curve of fluorescence intensity versus log10-transformed target-equivalent copy numbers, triangular symbols represent LFS results from crude lysate samples, while circular symbols represent LFS results from genomic DNA samples.

Reproducible low-intensity T-line signals were observed for *L. decempunctata* on both MIRA-LFS and RPA-LFS platforms. Signals were detected in two independently collected specimen batches and were observed with both crude lysates and purified genomic DNA templates (Figure 2). Based on regression models derived from plasmid standards, signal intensity corresponded approximately to 10^2^–10^3^ target-equivalent copies per reaction (Figure S3). These values represent order-of-magnitude estimates inferred from lateral flow signal intensity rather than absolute quantification.

Red boxes indicate representative T-line signals observed for *L. decempunctata*. Negative controls consisted of no-template controls prepared in the same dilution matrices (lysis buffer or ultrapure water).

No amplification was detected in the remaining 49 non-target species or in no-template controls. Sanger sequencing of selected amplification products did not identify *H. ligniperda*–specific sequences (Figures S4 and S5). The repeated detection of low-intensity signals for *L. decempunctata* across specimen batches, template types, and amplification platforms indicates that the observation was consistent under the tested conditions.

### 3.2. Quantitative Performance of SYBR Green qPCR

**Figure 3:**
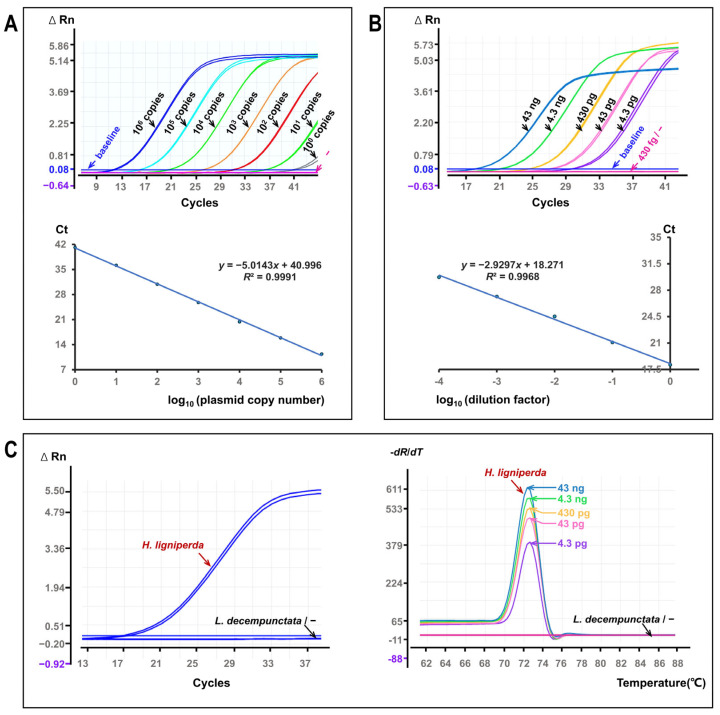
Quantitative performance of SYBR Green qPCR for *H. ligniperda*. (**A**) Amplification curves generated from tenfold serial dilutions of plasmid standards containing the *H. ligniperda* target fragment (ΔRn vs. cycle number). The corresponding standard curve shows threshold cycle (Ct) values plotted against log_10_-transformed plasmid copy number. (**B**) Amplification curves obtained from tenfold serial dilutions of *H. ligniperda* genomic DNA (ΔRn vs. cycle number). The corresponding standard curve shows Ct values plotted against log_10_-transformed dilution factors. (**C**) Amplification curves for genomic DNA from *H. ligniperda* and *L. decempunctata*, together with no-template controls (ultrapure water substituted for template) (ΔRn vs. cycle number). Melt curve analysis (−dF/dT vs. temperature) of *H. ligniperda* genomic DNA shows a single specific peak, with peak height decreasing as template concentration decreases, while maintaining assay specificity. Lines denote three technical replicates of the same sample and may not fully overlap.

The SYBR Green qPCR assay showed linear amplification across the tested concentration ranges for both plasmid and genomic DNA templates (Figure 3). Plasmid standards spanning six orders of magnitude yielded a linear standard curve (*R*^2^ = 0.9991) with an amplification efficiency of 58% (Figure 3A). Serial dilutions of genomic DNA, ranging from picogram to nanogram levels per reaction, also produced linear amplification (*R*^2^ = 0.9968) with low variability among technical replicates (Figure 3B).

Amplification curves for *H. ligniperda* displayed typical exponential kinetics, and melt-curve analysis revealed single, well-defined peaks (Figure 3C). In contrast, *L. decempunctata* samples and no-template controls remained at baseline fluorescence throughout the reaction. No exponential amplification curves or specific melt peaks were observed.

### 3.3. Specificity Validation and Confirmatory Analysis

**Figure 4:**
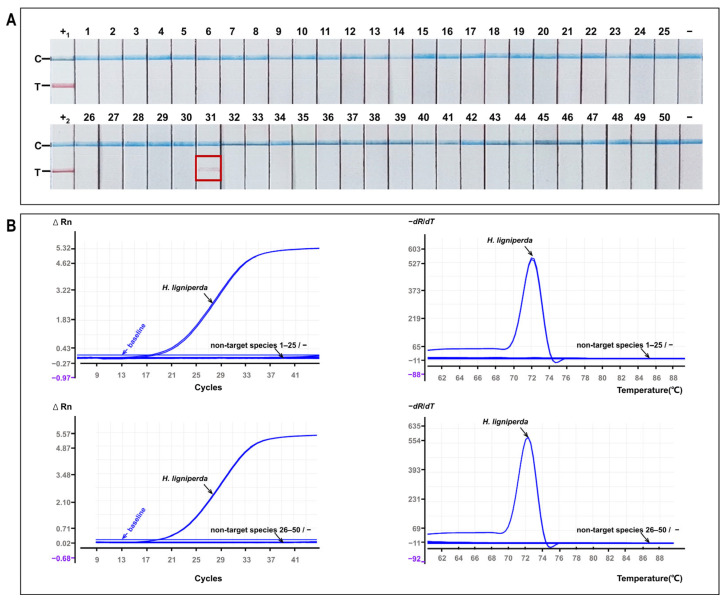
Specificity validation and confirmatory qPCR for border-intercepted arthropod species (*n* = 50). (**A**) MIRA-LFS results for all tested species, including *H. ligniperda* (target) and 49 non-target arthropods. Red boxes highlight low-intensity non-specific T-line signals only in *L. decempunctata* among 49 non-target arthropods. Negative controls (no-template controls prepared in the corresponding matrices) showed no signal. (**B**) SYBR Green qPCR analysis of the same sample set (*n* = 50) (ΔRn vs. cycle number). Amplification was observed only for *H. ligniperda*, while all non-target species, including *L. decempunctata,* and negative controls remained at baseline fluorescence.

The MIRA-LFS assay was applied to the complete panel of 50 non-target species (Figure 4A). Among these, only *L. decempunctata* produced reproducible low-intensity T-line signals. All other non-target species and negative controls remained negative.

All 50 non-target species were subsequently analysed using the SYBR Green qPCR assay to ensure direct comparability across platforms (Figure 4B). None of the non-target templates, including *L. decempunctata*, showed exponential amplification or specific melt peaks. Fluorescence signals remained at baseline levels throughout the reaction. These results indicate that non-target amplification was not supported by qPCR detection of the corresponding locus under the tested conditions.

## 4. Discussion

Molecular diagnostic assays for pest detection are traditionally validated using a taxonomy-centered specificity framework, where phylogenetically related species are prioritized for exclusivity testing. This approach assumes that evolutionary proximity and primer–template mismatches are the main drivers of non-specific amplification and has long served as the cornerstone for primer design and validation protocols [15,43]. While effective under controlled laboratory conditions, this framework may fall short in capturing the full range of performance challenges encountered in heterogeneous operational quarantine environments.

Our independent re-evaluation of a published isothermal detection system for *H. ligniperda* shows that evolutionary distance alone does not reliably predict assay behavior under trade-relevant conditions. Among fifty non-target arthropod species tested (predominantly insects), only the phylogenetically distant *L. decempunctata* produced weak but reproducible amplification signals. These signals were consistently detected across biological replicates, different template preparations (crude lysates and purified DNA), and two commercial isothermal platforms, pointing to a systematic analytical background rather than sporadic contamination or procedural artifacts.

In natural ecosystems, *H. ligniperda* and *L. decempunctata* occupy distinct ecological niches and are geographically isolated, with their adult stages readily distinguishable by morphology [32,44,45,46,47]. However, early developmental stages—such as eggs and larvae—of phylogenetically distant insect species often lack diagnostically informative morphological features, making reliable identification challenging. During international shipments of pine wood packaging materials containing goji berry products, these unrelated early life stages are frequently co-intercepted within the same consignment and processed as mixed samples. We term this operationally driven co-occurrence “logistics-mediated artificial sympatry.”

Logistics-mediated artificial sympatry does not itself induce molecular cross-reactivity; rather, it substantially increases the probability that non-target templates capable of low-level non-specific amplification (*L. decempunctata* DNA) will enter the quarantine screening process. Consequently, the risk of misinterpreting faint non-specific signals as positive for *H. ligniperda* is markedly elevated—particularly in mixed samples containing eggs or larvae, where morphological differentiation is not feasible.

The low-intensity signals observed are consistent with infrequent primer initiation events under the relatively relaxed stringency conditions inherent to isothermal amplification [48]. Agarose gel electrophoresis and cloning-based Sanger sequencing yielded no interpretable non-specific amplicons, and contamination was ruled out by appropriate controls. Although the precise molecular mechanism remains unresolved, the reproducibility across platforms and independent batches supports the interpretation of a consistent low-level analytical background under heterogeneous sample conditions. Quantitative estimation using plasmid standards indicated that these unintended signals corresponded to approximately 10^2^–10^3^ target-equivalent copies per reaction. In field-deployable rapid screening systems, signals of this magnitude can readily exceed positivity thresholds, especially when assays are applied beyond their original validation scope.

In practical quarantine settings, even rare ambiguous results can trigger precautionary regulatory responses under risk-averse biosecurity policies. These may include shipment detention pending confirmatory analysis, mandatory laboratory testing, temporary trade restrictions, or intensified inspection regimes. While protective in intent, such measures increase inspection workload, delay cargo clearance, and generate avoidable compliance costs. The cumulative impact can be substantial in high-throughput trade corridors where rapid decision-making is essential.

To mitigate this interpretive uncertainty, we evaluated a tiered diagnostic framework that integrates rapid on-site isothermal screening with laboratory-based SYBR Green qPCR confirmation. The qPCR assay was designed under a specificity-prioritized principle consistent with established recommendations for applied molecular diagnostics [42]. Given the potential regulatory consequences of false-positive results in logistics and border inspection contexts, an amplification enhancer was incorporated to reduce non-specific amplification rather than to maximize amplification kinetics. Despite a moderate amplification efficiency (58%), the assay exhibited strong linearity, a single well-defined melt peak, complete exclusivity across all non-target species tested, and superior analytical sensitivity relative to the isothermal method under identical conditions.

These results suggest that amplification efficiency, analytical sensitivity, and specificity should be considered jointly when evaluating diagnostic performance. Although amplification efficiency is often used as a general indicator, it does not directly determine either detection sensitivity or the ability to discriminate non-target templates. In practice, these parameters are interdependent and may involve trade-offs: conditions that enhance specificity can reduce amplification efficiency, while improved amplification kinetics do not necessarily improve assay selectivity [49].

In the present study, the qPCR assay was developed with an emphasis on specificity to ensure reliable interpretation under complex sample backgrounds. This resulted in moderate amplification efficiency but enabled consistent exclusion of non-target species and improved resolution of low-intensity signals. These observations indicate that, in quarantine and regulatory contexts, assay performance is better assessed by its ability to balance sensitivity and specificity under operational conditions rather than by amplification efficiency alone.

Within this two-tier workflow, isothermal amplification serves as a rapid, high-throughput primary screen, while qPCR provides confirmatory resolution for low-intensity or borderline-positive samples. This structure preserves operational efficiency while enhancing interpretive robustness. The framework holds potential applicability to other invasive pest detection programs, pooled-sample surveillance strategies, and broader biosecurity monitoring systems.

Collectively, our findings indicate that taxonomy-centered specificity validation alone may be insufficient when diagnostic assays are deployed in heterogeneous, trade-associated environments. Incorporating realistic operational sampling scenarios into validation protocols, together with confirmatory testing layers in diagnostic pipelines, is essential to enhance robustness, reduce interpretive ambiguity, and minimize unnecessary regulatory interventions in applied biosecurity contexts.

## Figures and Tables

**Table 1:** Primer sequences for SYBR Green I qPCR confirmation and published MIRA-LFS detection of *H. ligniperda*.

Primer		Sequence (5′→3′)	Product(bp)
SYBR Green I qPCR Assay	CLXD-F	CAGGGAGCCAGAAATGAAAGGGGG	237
	CLXD-R	ACTTATTATCTATTATTCCCTTAA	
MIRA-LFS Assay [15]	HLRPA-F	TCTCCTCTAGATCTTGATTAACCGCCTGAA	HLRPA-F/R: 273, HLRPA-P3/R: 208
	HLRPA-P3	[biotin]TTAATAAAATCCAATAAATACTTTTCTTCA(THF)AAACTATAACCAAAT[pho]	
	HLRPA-R	[FAM]AGAAATGAAAGGGGGATGCTCCTATTTTTA	

## Data Availability

The original contributions presented in this study are included in the article/Supplementary Material. Further inquiries can be directed to the corresponding author.

## Supplementary Materials

The following supporting information can be downloaded at: https://www.mdpi.com/article/10.3390/insects17040408/s1, Figure S1: Comparative morphology of the target and non-target species; Figure S2: Sequence alignment and validation of MIRA and qPCR amplicons within the mitochondrial genome; Figure S3: Analytical sensitivity of MIRA and RPA assays via lateral flow detection; Figure S4: Gel electrophoresis analysis of MIRA and RPA products; Figure S5: Sanger sequencing and identity validation of amplicons; Table S1: List of border-intercepted arthropod species (*n* = 50) utilized for risk-based diagnostic robustness assessment.

## Abbreviations

The following abbreviations are used in this manuscript:

MIRA	multiple enzyme isothermal rapid amplification
RAA	recombinase-aided amplification
RPA	recombinase polymerase amplification
LFS	lateral flow strips
