# Supplementary material for: Logistics-Mediated Artificial Sympatry and Its Implications for Molecular Detection of Hylurgus ligniperda
Source: Insects. 2026 Apr 9;17(4):408. doi: 10.3390/insects17040408 (PMC13117259; doi:10.3390/insects17040408)
Supplement: Supplementary file 1 [file insects-17-00408-s001.zip › insects-4210354-supplementary.pdf]

Supplementary Table S1. List of border-intercepted arthropod species (n = 50) utilized for risk-based diagnostic robustness assessment**.**

No.	Species	Order	Family (Subfamily)	Validation Tier
**1**	*Dendroctonus valens*	Coleoptera	Curculionidae (Scolytinae)	**Tier I** – High phylogenetic proximity
**2**	*Dendroctonus rufipennis*	Coleoptera	Curculionidae (Scolytinae)	**Tier I** – High phylogenetic proximity
**3**	*Ips grandicollis*	Coleoptera	Curculionidae (Scolytinae)	**Tier I** – High phylogenetic proximity
**4**	*Orthotomicus caelatus*	Coleoptera	Curculionidae (Scolytinae)	**Tier I** – High phylogenetic proximity
**5**	*Ips pini*	Coleoptera	Curculionidae (Scolytinae)	**Tier I** – High phylogenetic proximity
**6**	*Ips typographus*	Coleoptera	Curculionidae (Scolytinae)	**Tier I** – High phylogenetic proximity
**7**	*Orthotomicus erosus*	Coleoptera	Curculionidae (Scolytinae)	**Tier I** – High phylogenetic proximity
**8**	*Ips calligraphus*	Coleoptera	Curculionidae (Scolytinae)	**Tier I** – High phylogenetic proximity
**9**	*Xyleborus ferrugineus*	Coleoptera	Curculionidae (Scolytinae)	**Tier II** – Same order, different subfamily
**10**	*Xyleborus affinis*	Coleoptera	Curculionidae (Scolytinae)	**Tier II** – Same order, different subfamily
**11**	*Xyleborus germanus*	Coleoptera	Curculionidae (Scolytinae)	**Tier II** – Same order, different subfamily
**12**	*Xyleborus perforans*	Coleoptera	Curculionidae (Scolytinae)	**Tier II** – Same order, different subfamily
**13**	*Hapalogenius atakorae*	Coleoptera	Curculionidae	**Tier II** – Same order, different family
**14**	*Alphitobius diaperinus*	Coleoptera	Tenebrionidae	**Tier II** – Same order, different family
**15**	*Sitophilus oryzae*	Coleoptera	Curculionidae	**Tier II** – Same order, different family
**16**	*Monochamus* sp.	Coleoptera	Cerambycidae	**Tier II** – Same order, different family
**17**	*Nacerdes melanura*	Coleoptera	Oedemeridae	**Tier II** – Same order, different family
**18**	*Xystrocera globosa*	Coleoptera	Cerambycidae	**Tier II** – Same order, different family

No.	Species	Order	Family (Subfamily)	Validation Tier
**19**	*Mesosa irrorata*	Coleoptera	Cerambycidae	**Tier II** – Same order, different family
**20**	*Pygmodeon* sp. (Bolivia)	Coleoptera	Cerambycidae	**Tier II** – Same order, different family
**21**	*Pygmodeon* sp. (Cambodia)	Coleoptera	Cerambycidae	**Tier II** – Same order, different family
**22**	*Pygmodeon* sp. (China)	Coleoptera	Cerambycidae	**Tier II** – Same order, different family
**23**	*Bruchinae* sp.	Coleoptera	Chrysomelidae (Bruchinae)	**Tier II** – Same order, different family
**24**	*Ophraella communa*	Coleoptera	Chrysomelidae	**Tier II** – Same order, different family
**25**	*Basilepta fulvipes*	Coleoptera	Chrysomelidae	**Tier II** – Same order, different family
**26**	*Diabrotica virgifera*	Coleoptera	Chrysomelidae	**Tier II** – Same order, different family
**27**	*Chrysomelidae* sp.	Coleoptera	Chrysomelidae	**Tier II** – Same order, different family
**28**	*Alticinae* sp.	Coleoptera	Chrysomelidae (Alticinae)	**Tier II** – Same order, different family
**29**	*Psylliodes punctifrons*	Coleoptera	Chrysomelidae	**Tier II** – Same order, different family
**30**	*Elateridae* sp.	Coleoptera	Elateridae	**Tier II** – Same order, different family
**31**	*Lema decempunctata*	Coleoptera	Chrysomelidae	**Tier II** – Same order, different family
**32**	*Carabus* sp.	Coleoptera	Carabidae	**Tier II** – Same order, different family
**33**	*Cetoniinae* sp.	Coleoptera	Scarabaeidae (Cetoniinae)	**Tier II** – Same order, different family
**34**	*Ectatomma opaciventre*	Hymenoptera	Formicidae	**Tier III** – Different insect orders
**35**	*Solenopsis geminata*	Hymenoptera	Formicidae	**Tier III** – Different insect orders
**36**	*Formicidae* sp.	Hymenoptera	Formicidae	**Tier III** – Different insect orders
**37**	*Vespidae* sp.	Hymenoptera	Vespidae	**Tier III** – Different insect orders
**38**	*Pyralidae* sp.	Lepidoptera	Pyralidae	**Tier III** – Different insect orders
**39**	*Pieridae* sp.	Lepidoptera	Pieridae	**Tier III** – Different insect orders
**40**	*Acrididae* sp.	Orthoptera	Acrididae	**Tier III** – Different insect orders

No.	Species	Order	Family (Subfamily)	Validation Tier
**41**	*Coptotermes* sp.	Blattodea	Rhinotermitidae	**Tier III** – Different insect orders
**42**	*Bactrocera tau*	Diptera	Tephritidae	**Tier III** – Different insect orders
**43**	*Bactrocera dorsalis*	Diptera	Tephritidae	**Tier III** – Different insect orders
**44**	*Dacus trimacula*	Diptera	Tephritidae	**Tier III** – Different insect orders
**45**	*Asilidae* sp.	Diptera	Asilidae	**Tier III** – Different insect orders
**46**	*Chrysopidae* sp.	Neuroptera	Chrysopidae	**Tier III** – Different insect orders
**47**	*Cosmoscarta exultans*	Hemiptera	Cercopidae	**Tier III** – Different insect orders
**48**	*Erthesina fullo*	Hemiptera	Pentatomidae	**Tier III** – Different insect orders
**49**	*Pyrrhocoridae* sp.	Hemiptera	Pyrrhocoridae	**Tier III** – Different insect orders
**50**	*Araneidae* sp.	Araneae	Araneidae	**Tier IV** – Non-insect arthropods

Taxonomic classification follows the current international systematic consensus. Validation tiers were categorized based on phylogenetic proximity to the target species and the potential risk of diagnostic interference under operational border interception scenarios.

Note: To ensure rigorous exclusivity testing, all genomic DNA templates were standardized to a concentration of 40–50 ng·μL^{-1}.

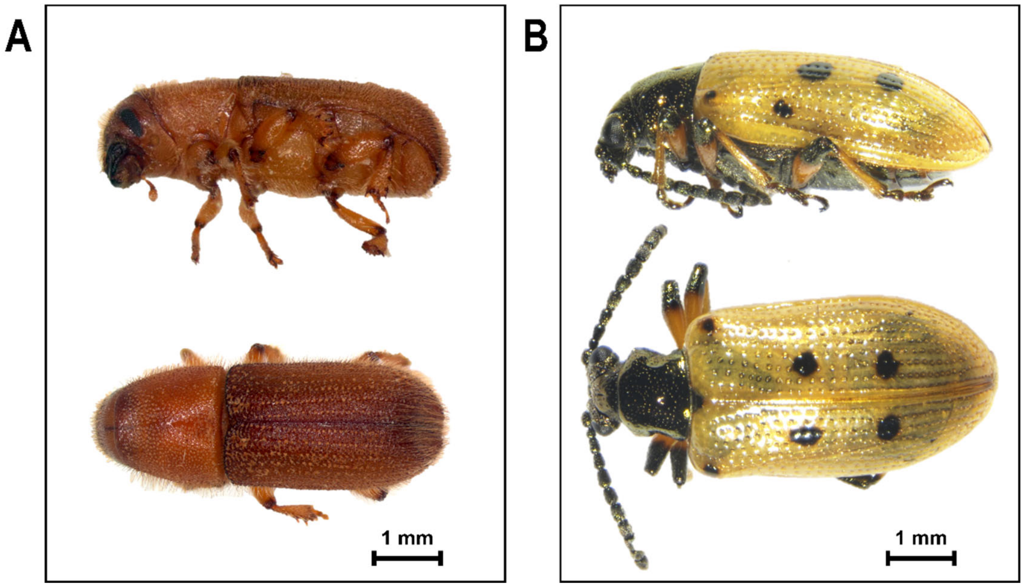

Supplementary Figure S1. Comparative morphology of the target and non-target species. Representative stereomicroscopic images of adult specimens examined in this study: (A) the target bark beetle, *H. ligniperda*; and (B) the non-target leaf beetle, *L. decempunctata*. These species occupy distinct ecological niches but were investigated due to potential co-occurrence in logistical chains.

```
                7421 ......7430......7440......7450......7460......7470......7480......7490
OR105874.1 7421 CAGGGAGCCAGAAATGAAAGGGGGATGCCCCTATTTTTAATAAAAGAGCAGATGTTGATAAAAAGGATAT
HLRPA-F/R     1 ---------AGAAATGAAAGGGGGATGCCCCTATTTTTAATAAAAGAGCAGATGTTGATAAAAAGGATAT
CLXD-F/R      1 CAGGGAGCCAGAAATGAAAGGGGGATGCCCCTATTTTTAATAAAAGAGCAGATGTTGATAAAAAGGATAT
consensus  7421 cagggagccAGAAATGAAAGGGGGATGCtCCTATTTTTAATAAAAGAGCAGATGTTGATAAAAAGGATAT

                7491 ......7500......7510......7520......7530......7540......7550......7560
OR105874.1 7491 TGGGGTTAAGAAGTTATTAATTAGGTTGAAATCTTTTAAATTAACTATTGTGATTAAAGAAAAAAGTAAA
HLRPA-F/R    62 TGGGGTTAAGAAGTTATTAATTAGGTTGAAATCTTTTAAATTAACTATTGTGATTAAAGAAAAAAGTAAA
CLXD-F/R     71 TGGGGTTAAGAAGTTATTAATTAGGTTGAAATCTTTTAAATTAACTATTGTGATTAAAGAAAAAAGTAAA
consensus  7491 TGGGGTTAAGAAGTTATTAATTAGGTTGAAATCTTTTAAATTAACTATTGTGATTAAAGAAAAAAGTAAA

                7561 ......7570......7580......7590......7600......7610......7620......7630
OR105874.1 7561 ATTATGGAAGAAATTGCTTGAATTATAAAATATTTGGTTATAGTTTCTGAAGAAAAGTATTTATTGGATT
HLRPA-F/R   132 ATTATGGAAGAAATTGCTTGAATTATAAAATATTTGGTTATAGTTTCTGAAGAAAAGTATTTATTGGATT
CLXD-F/R    141 ATTATGGAAGAAATTGCTTGAATTATAAAATATTTGGTTATAGTTTCTGAAGAAAAGTATTTATTGGATT
consensus  7561 ATTATGGAAGAAATTGCTTGAATTATAAAATATTTGGTTATAGTTTCTGAAGAAAAGTATTTATTGGATT

                7631 ......7640......7650......7660......7670......7680......7690......7700
OR105874.1 7631 TTATTAAGGGAATAATAGATAATAAGTTTATTTCTAGCCCTATTCAGGCGGTTAATCAAGATCTAGAGGA
HLRPA-F/R   202 TTATTAAGGGAATAATAGATAATAAGTTTATTTCTAGTCCTATTCAGGCGGTTAATCAAGATCTAGAGGA
CLXD-F/R    211 TTATTAAGGGAATAATAGATAATAAGT-------------------------------------------
consensus  7631 TTATTAAGGGAATAATAGATAATAAGTttatttctag cctattcaggcggttaatcaagatctagagga

                7701 ......7710......7720......7730......7740
OR105874.1 7701 GATAGAGATTAATCTTCTTAAAATTAGTATGGAATATAAT
HLRPA-F/R   272 GA--------------------------------------
CLXD-F/R        ----------------------------------------
consensus  7701 ga
```

Supplementary Figure S2. Sequence alignment and validation of MIRA and qPCR amplicons within the mitochondrial *ND2* gene. Alignment of the MIRA and SYBR Green qPCR amplification regions within the partial *ND2* gene sequence of *Hylurgus ligniperda* (GenBank OR105874.1). The schematic illustrates the overlapping diagnostic regions: the MIRA segment (defined by HLRPA-F/R, 7430–7702 bp) and the qPCR segment (defined by CLXD-F/R, 7421–7657 bp). The sequence trace (Sanger sequencing) of the experimental *H. ligniperda* amplicon is

aligned with the reference, confirming the target specificity of the developed assays.

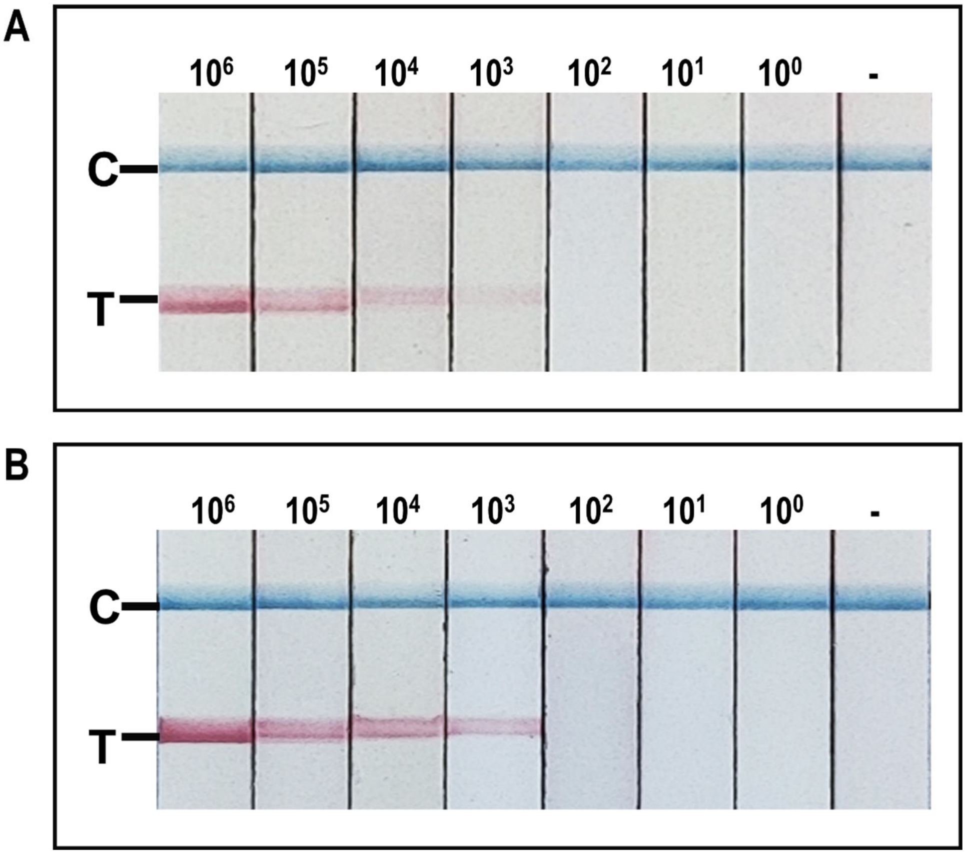

Supplementary Figure S3. Analytical sensitivity of MIRA and RPA assays via lateral flow detection. Representative lateral flow strips (LFS) showing the detection limits for serial tenfold dilutions of the *H. ligniperda* target plasmid: (A) MIRA-LFS assay and (B) RPA-LFS assay. The test (T) line remains visible down to 10^3 copies per reaction. Lower concentrations (10^2–10^0copies) and no-template controls (–) exhibit only the control (C) line, defining the visual detection thresholds for both platforms.

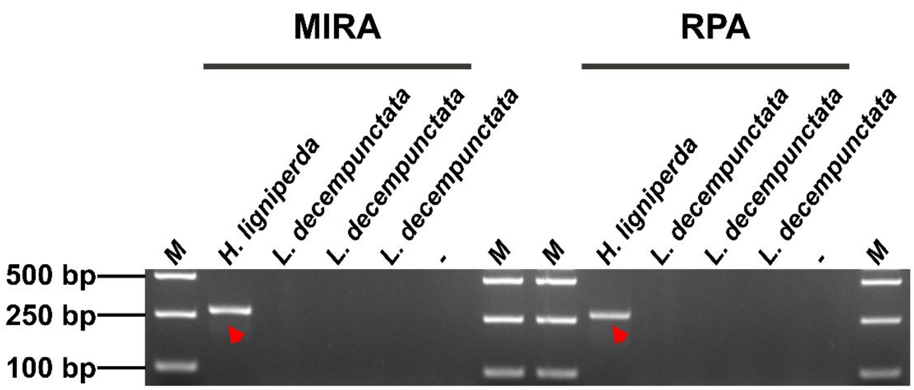

Supplementary Figure S4. Gel electrophoresis analysis of MIRA and RPA amplification products. Representative 2% agarose gel electrophoretic profiles showing amplification products using genomic DNA templates. Specific bands of the expected size (~273 bp) were consistently observed for *H. ligniperda* in both MIRA and RPA platforms (indicated by red arrows). No discernible specific bands were detected for *L. decempunctata* or the no-template controls (–), suggesting that weak LFS signals for the non-target species do not correspond to high-yield specific amplicons. M: DNA marker.

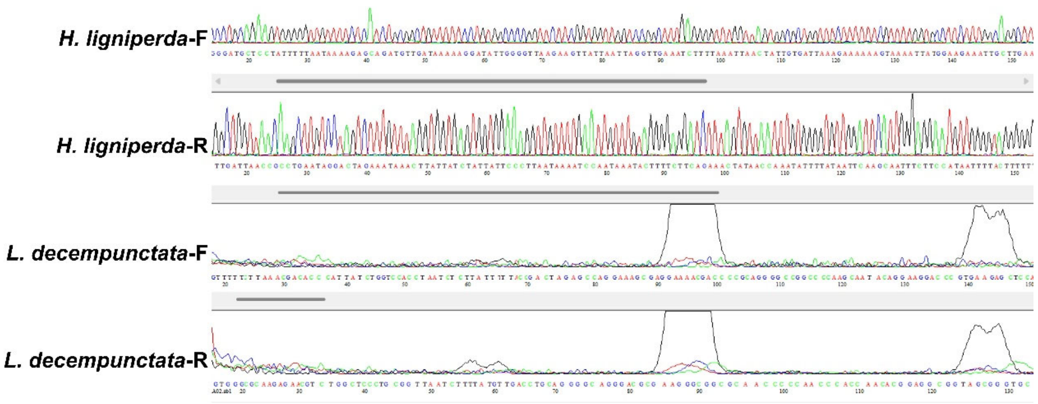

Supplementary Figure S5. Sanger sequencing and identity validation of amplicons.

Chromatogram representing the Sanger sequencing results of MIRA amplification

products. The *H. ligniperda* sequence showed 99.6% identity with the reference genome (GenBank OR105874.1), confirming target-specific diagnostic performance. In contrast, despite weak T-line signals in LFS detection, no interpretable sequencing data could be recovered from *L. decempunctata* samples. This confirms that the observed cross-reactivity does not stem from true target sequences or laboratory contamination.
